# Spontaneous Gastroduodenal Artery Hemorrhage Due to Median Arcuate Ligament Syndrome: A Case Report

**DOI:** 10.7759/cureus.105655

**Published:** 2026-03-22

**Authors:** Mitchell Shellman, Jared Mugfor

**Affiliations:** 1 Emergency Medicine, Allegheny Health Network, Pittsburgh, USA

**Keywords:** case report, gastroduodenal hemorrhage, massive blood transfusion, median arcuate ligament, unexplained syncope

## Abstract

Median arcuate ligament syndrome is a rare anatomic disorder leading to symptoms of foregut ischemia that can be complicated by aneurysm formation and rupture. This syndrome is difficult to diagnose because there is no direct study to evaluate it, and it is often discovered incidentally on imaging or following complications. Aneurysm rupture presents a life-threatening complication due to the volume of potential hemorrhage secondary to increased back pressure.

A 46-year-old male presented with an acute atraumatic onset of severe abdominal pain and was found to have a massive intra-abdominal hemorrhage due to gastroduodenal artery aneurysm rupture. Median arcuate ligament syndrome was diagnosed during operating room re-exploration. Following ligament resection and vessel ligation, the patient was stabilized and had an uneventful post-operative course. Median arcuate ligament syndrome is a rare condition that is a diagnosis of exclusion during the workup of chronic mesenteric ischemia. Complications of this syndrome are even more rare, with a gastroduodenal artery (GDA) aneurysm presenting as an even more rare but potentially more dangerous complication given the increased size of this proximal vessel. While rare, this case presents a very important reminder when assessing patients with long-standing vague abdominal symptoms, concern for chronic mesenteric ischemia, and the potential critical course resulting from this disease process, necessitating prompt intervention.

## Introduction

The median arcuate ligament is a fibrous connective tissue joining the diaphragmatic left and right crura at the aortic hiatus, forming the superior aspect of the hiatus proximal to the celiac artery. Median arcuate ligament syndrome (MALS), also known as Dunbar syndrome, first described in a 1963 case series, presents with vague symptoms of foregut ischemia secondary to celiac artery (CA) compression, and lacks a definitive diagnostic test. Foregut ischemic symptoms include abdominal discomfort following periods of increased enteric metabolism, notably post-prandial and with exercise, nausea, emesis, unintentional weight loss, and food aversion [1]. Following compression of the CA by the fibrous median arcuate ligament based on anatomical variation of the celiac trunk, the vasculature undergoes histologic changes, including smooth muscle proliferation, disorganization of the medial and adventitial layers, and abnormal elastic fiber [2]. MALS drastically increases the incidence of visceral aneurysms secondary to these changes. One study reported an increased visceral artery aneurysm rate of 48% compared to the general population rate of 0.1-2%, with 73% of identified aneurysms in the pancreaticoduodenal arcade, 27% in the splenic artery, and 18% in the CA [3]. Therefore, any patient diagnosed with or being evaluated for MALS should bear a high clinical suspicion for visceral artery aneurysms and the resulting pathology. The true incidence of MALS is difficult to ascertain, as retrospective studies rely on computed tomography angiography (CTA) imaging review; one study identified MALS in 2.8% of imaging, and only three patients endorsed symptoms consistent with MALS [4]. General consensus is that MALS has a 4:1 female to male predominance, presenting at a median age of 30-50 years [5]. Aneurysm development has been shown to result from retrograde flow volume and, subsequently, increased pressure to maintain perfusion in the setting of CA compression [6].

We present a case of previously undiagnosed MALS leading to spontaneous gastroduodenal artery (GDA) aneurysm rupture. The GDA is one of the first major arterial branches off the common hepatic artery, which in turn arises from the celiac artery. Given the rarity of this disease process and the aneurysm affecting GDA being an uncommonly reported site of MALS complication, we hope to raise awareness of this disease process and the severity of consequences.

## Case presentation

We present a case of a 46-year-old male patient with a history notable for hypertension, asthma, gastroesophageal reflux disease, irritable bowel syndrome, and ongoing abdominal pain for 10 years. He previously underwent esophagogastroduodenoscopy and colonoscopy in April 2015, which only demonstrated subtle nodularity in the second part of the duodenum. The patient endorsed persistent post-prandial nausea and vomiting, unchanged and unrelieved after undergoing cholecystectomy. He was maintained on 20 mg omeprazole daily, losartan-hydrochlorothiazide, nighttime gabapentin, and as-needed lorazepam with no history of tobacco use.

On the day prior to presentation, the patient had an acute atraumatic onset of severe generalized abdominal pain. At the time of presentation, he was noted to be diaphoretic and had experienced syncope, with a systolic pressure of 86 mmHg. Systolic pressure improved to 115 mmHg after receiving 2 L of intravenous fluid. On examination, the patient had diffuse abdominal tenderness and a second episode of syncope. Hemoglobin was initially recorded at 12.9 g/dL, with a repeat measurement dropping to 10.7 g/dL over an unspecified time frame. Other lab work was notable for hypokalemia to 2.6 mmol/L, for which the patient underwent intravenous potassium repletion. Due to contrast allergy, the patient underwent a non-contrast CT scan, significant for a 7.8x5.2 cm right upper quadrant oval hypodense lesion consistent with a hematoma. Table 1 presents the selected laboratory values of interest, along with their accompanying reference ranges.

**Table 1:** Selected laboratory values and reference ranges.

Laboratory test	Time	Value	Reference range
Hemoglobin (g/dL)	Outside hospital initial	12.9	12.3-15.3
	Outside hospital repeat (undocumented interval)	10.7	
	Post-transfer initial	13.4	
Platelets (×10³/µL)	Post-transfer initial	117	144-445
Potassium (mmol/L)	Outside hospital initial	2.6	3.5-5.2
	Post-transfer initial	2.9	
Creatinine (mg/dL)	Post-transfer initial	1.32	0.5-0.9

Given the concern for a large volume of intra-abdominal blood loss, the patient was pre-medicated for a computed tomography angiography (CTA) with plans to transfer to a facility with vascular surgery capabilities. As the patient was being transferred into the ambulance, he had a 30-40 s seizure with associated hypotension of 63 mmHg over palpation and tachycardia of 150 beats per minute (bpm), prompting further resuscitation. He received 6 units of packed red blood cells (PRBC) and 1 unit of whole blood with an improvement in pressure to 116 systolic mmHg and a heart rate of 100 bpm.

He was deemed stable for LifeFlight transfer and arrived with 1 unit of PRBC transfusing with a systolic pressure of 70 mmHg, labored breathing on 6 L of oxygen via nasal cannula, and sinus tachycardia of 137 bpm. Patient was diaphoretic with thready femoral pulses, cool extremities, and a distended and diffusely tender abdomen with positive guarding.

**Figure 1:**
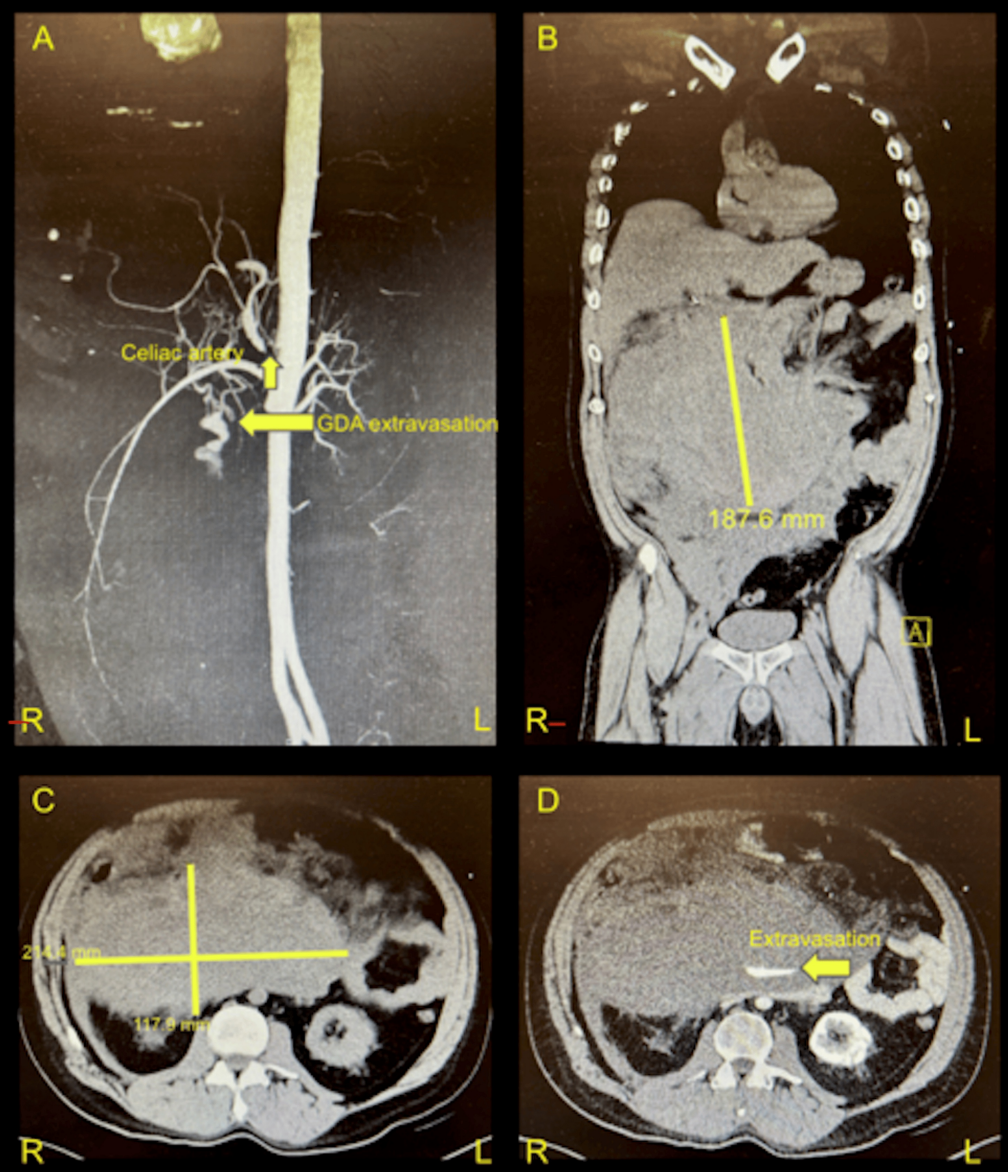
CTA of the abdomen and pelvis. (A) Vascular reconstruction demonstrating celiac trunk and active arterial extravasation. (B) Coronal view demonstrating initial hematoma with longitudinal expanse of 187.6 mm. (C) Axial view demonstrating hematoma with width of 214.4 mm and depth of 117.9 mm, approximate volume of 4.7 L. (D) Active contrast extravasation demonstrating hemorrhage into the posterior-medial aspect of the hematoma. CTA: computed tomography angiography

Initial laboratory values were notable for a hemoglobin level of 13.4 g/dL and thrombocytopenia with a platelet count of 117×10³/µL. Patient was noted to be persistently hypokalemic at 2.9 mmol/L with a new acute kidney injury with a creatinine of 1.32 mg/dL. CTA of the abdomen and pelvis demonstrated a massive volume intraperitoneal hematoma associated with hemoperitoneum with a large active bleed arising from a branch of the GDA or collateralized vessel from the left gastric wall. There was also noted severe to total stenosis of the celiac artery origin. Figures 1A-1D present key images from the CTA demonstrating these vascular pathologies and the extent of hemoperitoneum. A massive transfusion protocol was initiated, and the patient received 2 g of tranexamic acid, 1 unit of platelets, and 1 unit of packed red blood cells, with 40 μg of intravenous epinephrine administered for persistent hypotension. 

A second unit of packed red blood cells was transfused. He was administered 15 mg of ketamine to undergo placement of arterial and central lines in preparation for an emergent surgery. Given the amount of blood products the patient had received, he was also administered 1 g of calcium chloride. During transfer from the emergency department to the operating room, the patient required an additional 70 μg of intravenous epinephrine to maintain blood pressure.

In the operating room, a hemorrhaging GDA was successfully ligated, and an incidentally noted superior mesenteric vein branch with hemorrhage from the hematoma capsule was also successfully clipped and suture ligated. He did require 18 units of packed red blood cells and 18 units of fresh frozen plasma during this operative course. The patient was left with an open abdomen with a negative pressure wound dressing in place. Patient was transferred to the surgical ICU in stable condition with noted 1.2 L of bloody output from his negative pressure wound dressing within the first 3 h. The patient underwent thromboelastography-guided resuscitation, with further administration of 1 unit of packed red blood cells, 3 units of fresh frozen plasma, 2 units of cryoprecipitate, and 1 unit of platelets.

**Figure 2:**
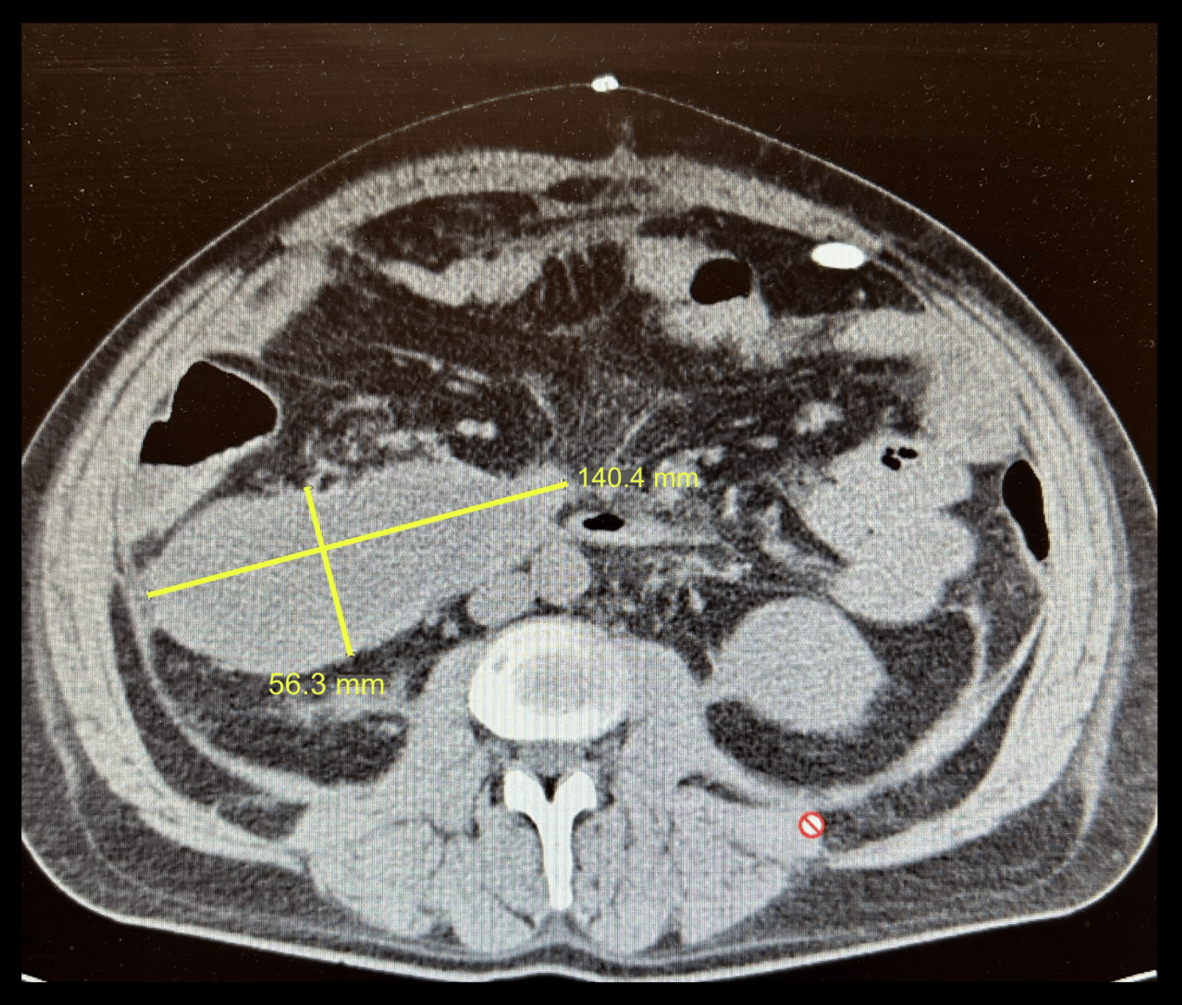
CT abdomen and pelvis demonstrating persistent intraperitoneal hematoma with significant volume reduction from initial presentation and imaging.

On post-operative day two, patient returned to the operating room for a median arcuate ligament and left diaphragmatic crus release after the celiac artery (CA) was identified to be compressed secondary to sclerotic tissue with no palpable pulse. Following the release of the ligament and crus, the celiac artery had a strong palpable pulse, and the abdomen was closed without complication, providing a clinical diagnosis of median arcuate ligament syndrome (MALS) given the response to intervention. Figure 2 presents repeat CTA of the abdomen and pelvis on post-operative day nine, which demonstrated a moderate reduction in size of the retroperitoneal hematoma with persistent CA ostia stenosis with approximately 90% narrowing. As the patient had resolution of symptoms and the celiac artery had a strongly palpable pulse following ligament and crus release intra-operatively, this imaging finding was considered not clinically relevant. Patient was successfully discharged on post-operative day 13.

## Discussion

This case had several interesting aspects. First, the aneurysm rupture is presumed to have occurred while the patient was at a low stress family function with no associated trauma or identification of factors that would acutely increase blood pressure leading to aneurysm rupture.

Second, the gastroduodenal artery (GDA) lies proximally within the collateral pathway between the superior mesenteric artery and the celiac artery and is typically larger in caliber than the pancreaticoduodenal arteries. However, aneurysm formation in median arcuate ligament syndrome (MALS) is better explained by altered hemodynamics rather than vessel size alone. Compression of the celiac artery increases retrograde collateral flow through the pancreaticoduodenal arcade and the GDA, exposing these vessels to chronically elevated flow and shear stress, which may promote aneurysmal degeneration. Although aneurysms most commonly arise in the pancreaticoduodenal arteries, the GDA remains part of this high-flow collateral network and therefore represents a plausible, albeit uncommon, site of aneurysm formation.

A case report describing spontaneous left gastroepiploic artery hemorrhage illustrates the rarity of vascular complications of MALS outside the pancreaticoduodenal arcade while highlighting the broader collateral circulation exposed to these altered hemodynamics [7]. In another report of an inferior pancreaticoduodenal artery aneurysm rupture, the lesion was successfully treated with coil embolization followed by delayed surgical release of the median arcuate ligament [8]. Whether similar minimally invasive approaches are feasible for GDA aneurysms is less certain, particularly in patients presenting in extremis with hemorrhagic shock. Another report describing hemorrhage from either the inferior GDA or pancreaticoduodenal artery also involved delayed diagnosis but resulted in a smaller retroperitoneal hematoma, suggesting that clinical severity may vary based on the precise anatomic location of aneurysm formation within the collateral network [9]. Management strategies remain debated. Some authors caution against GDA coil embolization in MALS because the vessel may serve as a critical collateral supplying the foregut in the setting of celiac artery stenosis [10]. Conversely, other reports describe successful endovascular embolization as a temporizing measure prior to definitive surgical release of the median arcuate ligament [11]. Further study is needed to clarify how the degree of celiac artery stenosis, collateral flow patterns, and aneurysm location influence optimal treatment strategies. In our case, the patient presented with a large intraperitoneal hematoma, whereas other reports describe upper gastrointestinal or retroperitoneal hemorrhage depending on the anatomic relationship of the aneurysm to the duodenum, pancreas, and retroperitoneal space.

Third, although the patient underwent recommended studies in working up chronic mesenteric ischemia, namely EGD and colonoscopy, he remained undiagnosed at the time of presentation with symptoms attributed to irritable bowel syndrome. On chart review, it is unclear whether the patient had ever undergone CT imaging during his workup of abdominal complaints, as this information was not available in the electronic medical record. However, as he had undergone cholecystectomy, the patient presumably had imaging that would have included his celiac trunk and potentially identified a narrowing consistent with MALS. This serves to demonstrate the complexity of the diagnosis of this rare condition, which ultimately was diagnosed intra-operatively.

A major limitation of this study is the lack of access to information from the initial hospital presentation. The timing of lab values, administration of interventions, and images obtained are unable to be accessed from the initial hospital, which limits the true demonstration of the critical timing of clinical decompensation. In addition, the lack of access to preceding records limits the assessment of prior history and evaluation of the patient's chronic abdominal pain, which could have provided further information that the source was undiagnosed MALS. With the provided information, MALS was diagnosed intra-operatively based on the improved blood flow following the empiric surgical release of the ligament and crus, providing retrospective data that indicates the most likely underlying etiology of the patient's symptoms and clinical course. This case report helps to raise awareness of a rare disease pathology with severe complications and encourages the consideration of advanced diagnostic evaluation when presented with a patient with vague chronic abdominal complaints. The severity of this case likely results from the involvement of the GDA and the higher hemorrhagic potential of this more proximal artery, though further case reports and studies would be needed in order to provide guided risk stratification of MALS based on visceral arterial aneurysm location.

## Conclusions

MALS is a rare condition that is a diagnosis of exclusion during the workup of chronic mesenteric ischemia. Complications of this syndrome are even more rare, with the pancreaticoduodenal artery (PDA) being the vascular arcade most affected by aneurysm formation, and the GDA aneurysm presenting as an even more rare but potentially more dangerous complication, given the increased size of this more proximal vessel. While rare, this case presents a very important reminder when assessing patients with long-standing vague abdominal symptoms, concern for chronic mesenteric ischemia, and the potential critical course resulting from this disease process, necessitating prompt intervention. Given the rarity of this disease and the severity of complications, further investigation into pursuing advanced or invasive diagnostic modalities for patients presenting with chronic abdominal symptoms in order to obtain diagnosis prior to emergent decompensation is warranted.
